# T1 signal intensity ratio correlation with T1 mapping in pediatric pancreatitis

**DOI:** 10.1007/s00261-024-04609-w

**Published:** 2024-10-01

**Authors:** Pradipta Debnath, Jean Tkach, Michelle Saad, David S. Vitale, Maisam Abu-El-Haija, Andrew T. Trout

**Affiliations:** 1 Cincinnati Children’s Hospital Medical Center, Cincinnati, USA; 2 University of Cincinnati, Cincinnati, USA

**Keywords:** Pancreas, Pancreatitis, MRI, T1, Signal intensity, Relaxometry

## Abstract

**Purpose:**

Our primary purpose was to understand the correlation between pancreas T1-weighted signal intensity ratio (SIR) and T1 relaxation time in children. We also sought to characterize differences in T1 SIR between children without and with pancreatitis.

**Methods:**

Retrospective study of patients < 18-years-old. SIR-pancreas:spleen (SIR-PS) and SIR-pancreas:paraspinal muscle (SIR-PM) were generated from T1-weighted gradient recalled echo images. Subdivided by field strength, T1 SIR was correlated (Spearman’s) with T1 relaxation time.

**Results:**

220 participants were included, 144 imaged at 1.5T (mean: 11.4 ± 4.2 years) and 76 imaged at 3T (mean: 10.9 ± 4.5 years). At 1.5T, SIR-PS (rho=-0.62, 95% CI: -0.71 to -0.51, *p* < 0.0001) and SIR-PM (rho=-0.57, 95% CI: -0.67 to -0.45, *p* < 0.0001) moderately negatively correlated with T1 relaxation time. At 3T, correlations between T1 SIR and T1 relaxation time were moderate (rho=-0.40 to -0.43, *p* ≤ 0.0003). SIR-PS was significantly different between patient groups at 1.5T (*p* < 0.0001) with pairwise differences between: normal vs. acute on chronic pancreatitis (1.52 vs. 1.13; *p* < 0.0001). SIR-PM was also significantly different between groups at 1.5T (*p* < 0.0001) with differences between: normal vs. acute pancreatitis (1.65 vs. 1.40; *p* = 0.0006), normal vs. acute on chronic pancreatitis (1.65 vs. 1.18; *p* < 0.0001), and normal vs. chronic pancreatitis (1.65 vs. 1.52; *p* = 0.0066). A SIR-PS cut-off of ≤ 1.31 had 44% sensitivity and 95% specificity and SIR-PM cut-off of ≤ 1.53 had 69% sensitivity and 70% specificity for pancreatitis. At 3T, SIR-PS was significantly different between groups (*p* = 0.033) but without significant pairwise differences.

**Conclusion:**

At 1.5T pancreas T1 SIR moderately to strongly correlates with estimated T1 relaxation time and is significantly lower in children with pancreatitis.

## Introduction

The incidence of acute pancreatitis and chronic pancreatitis in children are reported to be 12 and 2 per 100,000 persons per year respectively [1]. Despite a lower frequency of disease compared to adults, disease burden is still substantial in the pediatric population [2]. According to the **In**ternational **S**tudy group of **P**ediatric **P**ancreatitis: **I**n search for a cu**re** (INSPPIRE) consortium, imaging findings are an important component of diagnosis of both acute and chronic pancreatitis in children a [3].

One of the multiple magnetic resonance imaging (MRI) findings of pancreatitis is decreased signal intensity on T1-weighted (T1W) images, reflecting an increase in T1 relaxation time. This signal loss reflects edema in the context of acute pancreatitis and acinar cell loss in the context of chronic pancreatitis and can be identified subjectively, but interobserver agreement is only fair [4]. Quantitative T1 mapping, which provides estimated T1 relaxation time values, has been shown to be useful in objectively diagnosing pancreatitis in adults [5, 6]. T1 relaxation time estimates have also been reported to be different between children with normal pancreases and with acute or chronic pancreatitis [7]. T1 mapping requires specialized imaging techniques, and when performed in the abdomen, typically involves multiple breath holds. Thus, it may not be widely available or frequently used in children.

As an alternative to T1 mapping, the pancreas T1 signal intensity ratio (T1 SIR) is an objective quantitative measure of relative pancreas T1W signal intensity derived by normalizing the signal intensity of the pancreas to another structure on routine T1W MR images. T1 SIR can be derived from routine T1W MR imaging. Because T1W imaging is commonly acquired during abdominal MRI/MRCP and is considered part of a minimum MRI protocol for pediatric pancreatitis [8], it is theoretically more widely available than T1 mapping. Further T1W imaging can be obtained without breath holds through radial imaging techniques facilitating acquisition in children. The T1 SIR has been reported to be associated with the stage of chronic pancreatitis in a large multicenter prospective study in adults [9, 10] and has been shown to predict pancreatic fistula after pancreatic surgery in adults [11]. To our knowledge, there is only one prior study of pancreas T1 SIR in children [12]. This study reported normal values and cutoffs for the pancreas:spleen T1-weighted signal intensity ratio in children without pancreatic disease [12].

There is limited literature comparing T1 SIR with T1 relaxation time characterized using T1 mapping in either children or adults to understand the relationship between these different but related metrics. Therefore, the primary purpose of this study was to correlate T1 SIR with T1 relaxation time estimates in children. We also sought to characterize differences in T1 SIR between children with acute pancreatitis, chronic pancreatitis, acute on chronic cases of pancreatitis and no pancreas pathology.

## Methods

Under institutional review board approval, data were retrospectively collected at Cincinnati Children’s Hospital Medical Center a quaternary pediatric hospital. All study activities were HIPAA compliant. Consecutive patients who underwent a clinically indicated MRCP between January 2020 and August 2023 were identified using an imaging report search engine (Illuminate InSight v4.3, Softek Illuminate). From this sample, we included pediatric patients less than 18 years of age and only patients imaged on Philips MRI scanners (Philips Healthcare, Best, The Netherlands). The scanner manufacturer limitation was applied to avoid potential confounding based on technical differences in implementation of T1 mapping between MRI manufacturers.

Given the known dependence of T1 relaxation times on magnetic field strength, identified patients were sub-divided based on the field strength of scanners used (i.e., 1.5T, 3T). We excluded examinations without a visible and measurable pancreas and included no more than 12 patients per year of age for 1.5T scans, and no more than 6 patients per year of age for 3T scans, selecting the most recently performed scans in each age group. We applied this limitation to the number of patients included in each age group because of the potential that T1 relaxation time and, correspondingly, T1 SIR could change based on patient age. The selected group of patients have previously been reported, in a paper reporting T1 relaxation time estimates but not T1 SIR [7]. This work builds upon that previous work by exploring the potentially more available T1 SIR in children and exploring associations between T1 SIR and T1 relaxation time.

### MRI examinations

MRI examinations included in our study were obtained on one of the following MRI scanners: Philips Ingenia 1.5T, Philips Ingenia 3T, and Philips Ambition 1.5T (Philips Healthcare, Best, The Netherlands). These examinations included both T1 mapping and 3D T1 modified DIXON sequences which were compared for this study.

As detailed in our prior publication, T1 mapping was accomplished using an Electrocardiogram (ECG)-triggered two-dimensional Modified Look-Locker Inversion Recovery (MOLLI) sequence implemented as a (5 s(3 s)3 s) scheme using a balanced steady state free precession (bSSFP) gradient echo acquisition [7]. Parametric maps of T1 relaxation time, estimated based on a single compartment model, with 95% confidence maps overlaid, were generated immediately on the scanner console using the vendor’s product software (PACS) (Merge PACS, version 7.2.0.157991, Merge Healthcare).

T1 SIR metrics were generated from signal intensity measurements made on T1W MR images acquired using either a navigator gated 3-dimensional (3D) pseudo-golden-angle radial stack-of-stars chemical shift encoded (also known as mDIXON) radiofrequency (RF) spoiled gradient echo acquisition (3D VANE) [13–16]; or a single breath-hold 3D T1 mDIXON RF spoiled gradient echo acquisition. Both 3D acquisitions were acquired in the axial plane and positioned to cover the liver dome to the iliac crest. For both sequences, the water only image was used for the signal intensity measurements from which the T1 SIRs were calculated. Representative acquisition parameters for the 3D VANE sequence included: repetition time (TR) = 7.3 ms, echo time (TE) (*N* = 2) = 2.0 ;4.4ms, flip angle = 12 degrees; field of view (FOV), 320 × 320mm^2^; matrix, 320 × 320, turbo field echo (TFE) factor = 38, Radial % = 200, (SENSitivity Encoding) SENSE acceleration factor (in plane phase encode/slice phase encode) = 1 / 2.2, Half Fourier (slice) = 0.8, slice thickness = 4 mm; acquisition time ≥ 3:46 (navigator gated used for respiratory compensation). Representative acquisition parameters for the breath-hold T1 mDIXON RF spoiled gradient echo sequence: TR = 5.3 ms, TE (*N* = 2) = 1.74;3.6 ms, flip angle = 15 degrees; FOV, 320 × 320mm^2^; matrix, 212 × 212, SENSE acceleration factor (in plane phase encode/slice phase encode) = 2/ 1.8, slice thickness, 4 mm, Half Fourier (In plane Phase encode/Slice) = 0.675/0.75; acquisition time: ~16–17 s.

### Image analysis

Measurement of estimated T1 relaxation times has already been described for this study sample [7]. Briefly, a research fellow used a vendor-neutral post-processing platform (IntelliSpace, Philips Healthcare) to draw freehand regions of interest (ROI) on the T1 parametric map for each of the four slices, encompassing as much of the pancreas as possible on each slice. Mean T1 relaxation time was recorded for each ROI with an overall mean T1 relaxation time calculated as an area weighted mean of all ROI values.

**Fig. 1:**
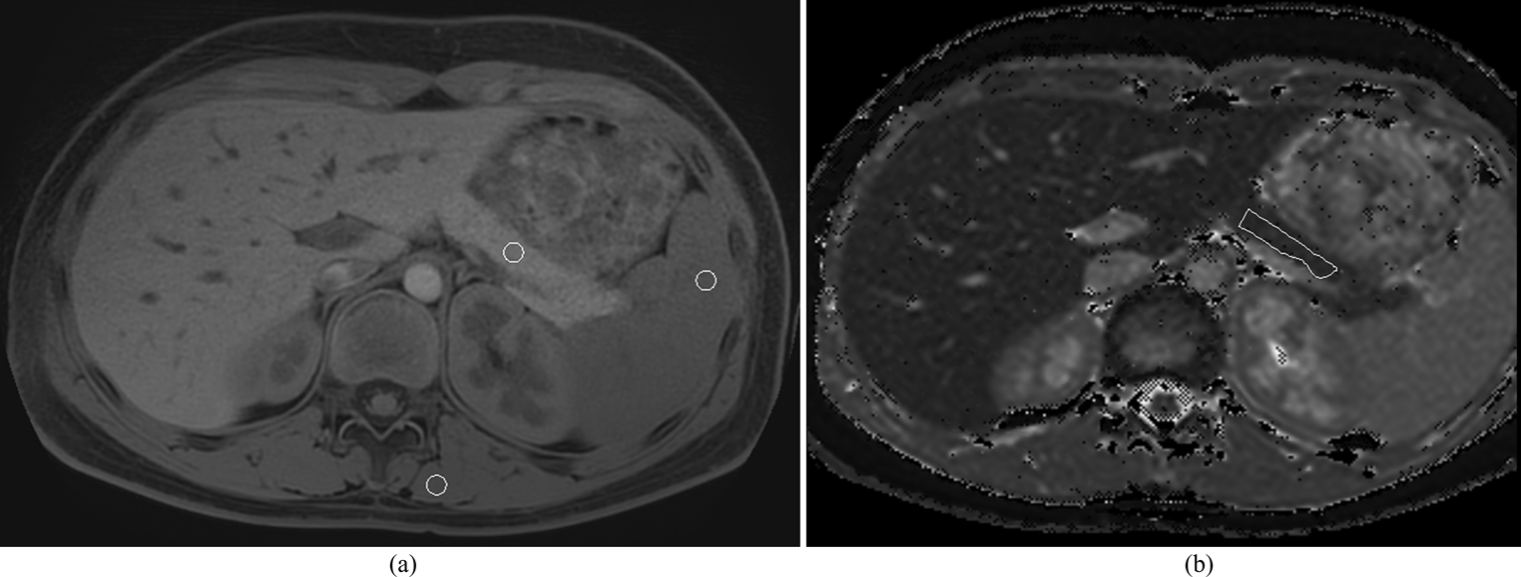
Axial 1.5T **(A)** T1-weighted 3D VANE and **(B)** T1 parametric map images in a 14-year-old boy with no pancreatic disease. Circular regions of interest (ROIs) in the pancreas, spleen, and paraspinal muscle on T1-weighted 3D VANE images were used to calculate T1-weighted signal intensity ratios (T1 SIR). An irregular ROI drawn on the pancreas on the corresponding slice on the T1 parametric map was used to measure estimated T1 relaxation time. ROIs are indicated using a white outline. Pancreas:spleen T1 SIR was 1.48 and Pancreas:paraspinal muscle T1 SIR was 1.70 with corresponding T1 relaxation time of 658 msec

**Fig. 2:**
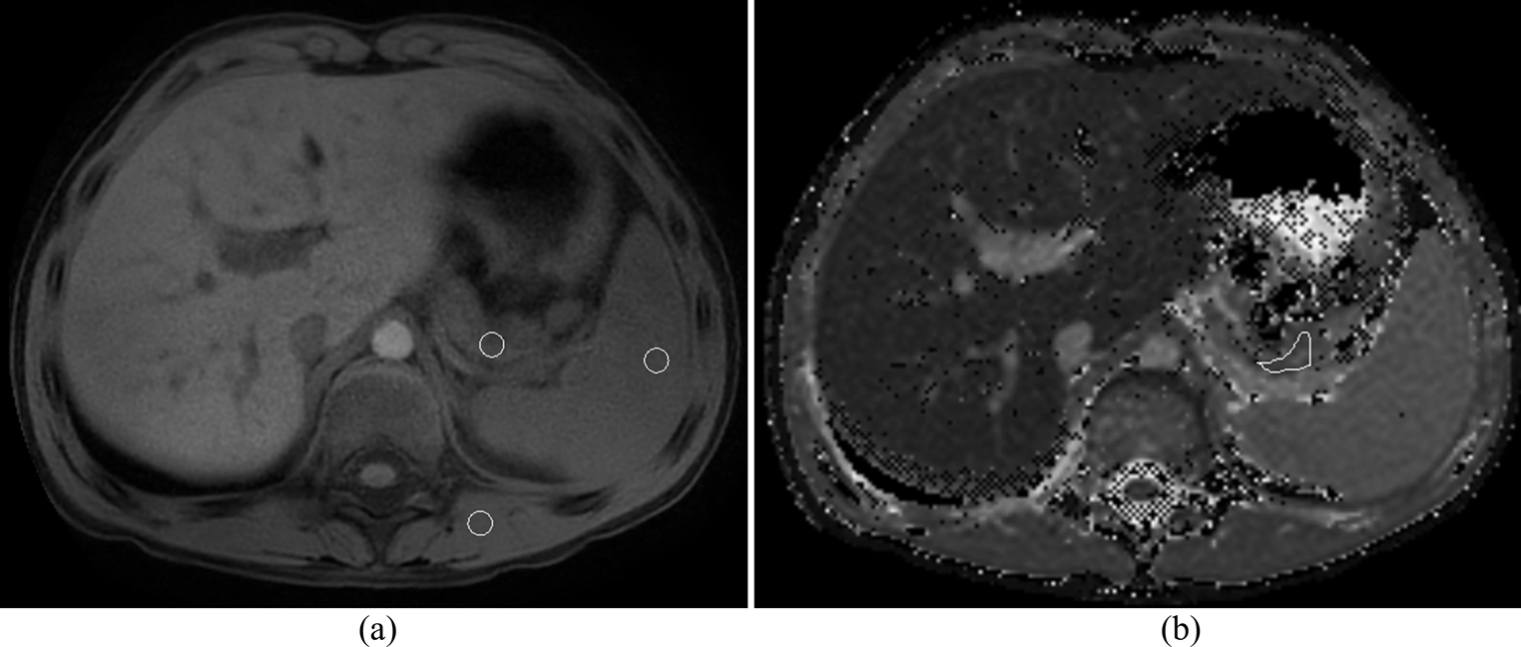
Axial 1.5T **(A)** T1-weighted 3D VANE and **(B)** T1 parametric map images in a 7-year-old boy with chronic pancreatitis. Circular regions of interest (ROIs) in the pancreas, spleen, and paraspinal muscle on T1-weighted 3D VANE images were used to calculate T1 weighted signal intensity ratios (T1 SIR). An irregular ROI drawn on the pancreas on the corresponding slice on the T1 parametric map was used to measure estimated T1 relaxation time. ROIs are indicated using a white outline. Pancreas:spleen T1 SIR was 1.02 and Pancreas:paraspinal muscle T1 SIR was 0.93 with corresponding estimated T1 relaxation time of 1053 msec

In addition, for the current study, at an interval of 90 days after the initial estimated T1 value measurements were made, the same research fellow measured signal intensities of the pancreas, spleen and paraspinal muscle on the single slice where the largest amount of pancreas was visible. Single, size-matched, ovoid ROIs were drawn in the pancreas, spleen and paraspinal muscle on either the navigator gated mDIXON 3D VANE images, or if not available, on the 3D T1 mDIXON RF spoiled gradient echo images (Figs. 1 and 2). Pancreas and spleen ROIs were drawn as close as possible to a similar anteroposterior level within the patient. Paraspinal muscle ROIs were drawn on the left in all cases.

All ROIs for measurement of T1 relaxation time (T1 maps) and T1 signal intensities were drawn by a single research fellow, experienced in analysis of pancreatic imaging. ROIs were reviewed and corrected as needed by a board-certified pediatric radiologist with more than 11 years of post-fellowship experience. T1 relaxation time estimates were reported as an ROI area weighted mean. Pancreas-to-spleen T1 SIR (SIR-PS) and pancreas-to-muscle T1 SIR (SIR-PM) were calculated using the following formula [10]:

$$\:T1\:SIR\:=\:\frac{SI\:Pancreas}{SI\:Reference}$$

Reference = spleen or paraspinal muscle.

### Medical record review

For all included patients, electronic health records (Epic; Verona, WI) were reviewed to record demographics, anthropometric data, and any relevant health conditions (involving the liver, spleen, pancreas). The radiology reports were also reviewed to identify the indications and imaging findings relevant to the pancreas (including presence of acute and chronic pancreatitis).

### Patient grouping

Patient subgroups were defined based on review of imaging reports, electronic medical records, and laboratory values. Additionally, relevant laboratory values were recorded which included amylase and lipase within 24 h of imaging date. Acute pancreatitis was defined by meeting at least two of three INSPPIRE diagnostic criteria (imaging findings, lipase ≥ 3x the upper limit, or abdominal pain) [3]. Chronic pancreatitis was defined also based on the INSPPIRE criteria which included imaging findings (ductal and/or parenchymal changes) plus one of the following: abdominal pain in the pancreatic region, exocrine insufficiency, or endocrine insufficiency [3]. The acute on chronic pancreatitis group met INSPPIRE criteria for both acute and chronic pancreatitis at the time of imaging. The control group was a group with no pancreas pathology, and no pancreatitis documented in their chart or evident on any prior imaging.

### Statistical analysis

Means and standardized deviations or medians and interquartile ranges (IQR) were used to summarize parametric and non-parametric continuous data respectively. Counts and percentages were used to summarize categorical data.

Because study data were non-normally distributed, non-parametric tests were used for statistical analysis. Spearman’s rank correlation coefficients were calculated between T1 SIR, and estimated T1 relaxation time measured on the same image and between T1 SIR and overall mean estimated T1 weighted relaxation time. The strength of correlation was classified as follows: 0-0.19, very weak; 0.2–0.39, weak; 0.40–0.59, moderate; 0.60–0.79, strong; and 0.80-1.0, very strong [17].

The Mann-Whitney U test was used to compare SIR between male and female patients while Spearman’s rank correlation coefficient correlation was used to correlate age with SIR values. The Kruskal–Wallis test was used to compare T1 SIR between subgroups with Dunn’s test used for pairwise comparisons.

Receiver operating characteristic (ROC) curve analysis was used to assess the use of T1 SIR to identify the presence of any pancreatitis (acute, chronic, or acute on chronic) compared to no pancreatic disease. Youden’s index was used to identify the threshold value that maximized sensitivity and specificity. A p value < 0.05 was considered statistically significant. Statistical analyses were performed using MedCalc Statistical Software version 22.009 (MedCalc Software Ltd., Ostend, Belgium).

## Results

We included a total of 220 patients in our study, 144 of whom had been imaged at 1.5T, and 76 of whom had been imaged at 3T. Relevant information regarding patient demographics, imaging indications and pancreas grouping are summarized in Table 1.

**Table 1:** Demographics and diagnosis grouping of pediatric patients undergoing magnetic resonance imaging at 1.5T and 3T field strengths

Variable	1.5T (*n* = 144) *n* (%)	3T (*n* = 76) *n* (%)
Age (Years)*	11.4 ± 4.2	10.9 ± 4.5
Sex	F = 76 (52.8%) M = 68 (47.2%)	F = 38 (50.0%) M = 38 (50.0%)
T1 sequence used for SIR	Radial T1 GRE = 119 (82.6%) 3D mDixon GRE = 25 (17.4%)	Radial T1 GRE = 72 (94.7%) 3D mDixon GRE = 4 (5.3%)
Indication for imaging	Pancreatitis = 79 (54.8%) Exocrine pancreatic insufficiency = 11 (7.6%) Choledochal cyst = 6 (4.2%) Biliary duct dilation = 5 (3.5%) Elevated liver enzymes = 5 (3.5%) Abdominal pain = 4 (2.8%) Autoimmune hepatitis = 4 (2.8%) Evaluate pancreas = 4 (2.8%) Other (each *n* < 4) = 26 (18.0%)	Pancreatitis = 53 (69.8%) Evaluate pancreas = 3 (3.9%) Abdominal pain = 2 (2.6%) Biliary duct dilation = 2 (2.6%) Evaluation for Total Pancreatectomy with Islet Autotransplantation = 2 (2.6%) Trauma = 2 (2.6%) Other (each *n* < 2) = 12 (15.9%)
Pancreas diagnosis grouping	Acute pancreatitis = 18 (12.5%) Acute on chronic pancreatitis = 19 (13.2%) Chronic pancreatitis = 28 (19.4%) No pancreas pathology = 79 (54.9%)	Acute pancreatitis = 19 (25.0%) Acute on chronic pancreatitis = 15 (19.7%) Chronic pancreatitis = 15 (19.7%) No pancreas pathology = 27 (35.6%)

F = female; M = male; SIR = signal intensity ratio

*Results presented as means and standard deviation

### T1 SIR correlations

**Fig. 3:**
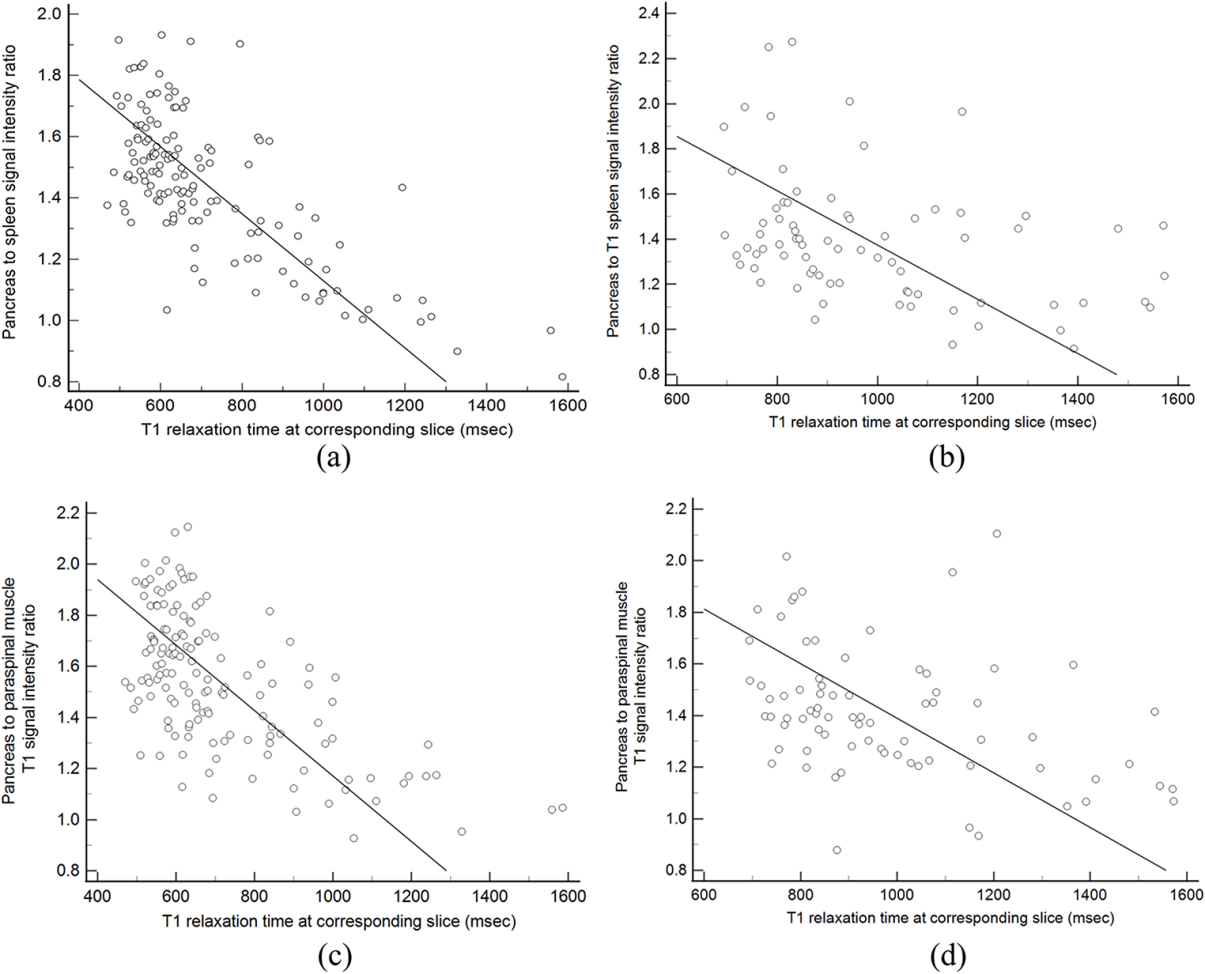
Scatter plots of pancreas to spleen T1 weighted signal intensity ratios (T1 SIR-PS) and T1 relaxation time estimates (msec) on the corresponding slice at **(A)** 1.5T and **(B)** 3T, and pancreas to paraspinal muscle T1 weighted signal intensity ratios (T1 SIR-PM) and T1 relaxation time estimates (msec) on the corresponding slice at **(C)** 1.5T and **(D)** 3T

At 1.5T, T1 SIR-PS was strongly negatively correlated with estimated T1 relaxation time for the same slice (rho = -0.62, 95% CI: -0.71 to -0.51) as well as with the ROI area weighted mean of the estimated T1 relaxation time across all images (rho = -0.63, 95% CI: -0.72 to -0.52) (Fig. 3). Similarly, T1 SIR-PM was moderately correlated with the estimated T1 relaxation time measured on the same slice (rho = -0.57, 95% CI: -0.67 to -0.45) and as well as with the ROI area weighted mean T1 relaxation time estimate across all images (rho = -0.54, 95% CI: -0.65 to -0.41). At 3T, correlations between T1 SIR and T1 relaxation time estimates were moderate (rho = -0.40 to -0.43) (Table 2).

**Table 2:** Spearman (rho) correlations between estimated T1 relaxation time and pancreas:spleen or pancreas:paraspinal muscle T1 weighted signal intensity ratios (T1 SIR). Results are presented for T1 SIR and T1 relaxation time measurements made on the same slice and for T1 SIR vs. an ROI area weighted mean T1 relaxation time estimate across all T1 parametric map images

Field Strength	T1 SIR	T1 relaxation time estimate on corresponding slice rho, (95% CI), *P* value	ROI area weighted mean T1 relaxation time estimate rho, (95% CI), *P* value
1.5T	pancreas:spleen	-0.62 (-0.71 to -0.51) *p* < 0.0001	-0.63 (-0.72 to -0.52) *p* < 0.0001
	pancreas:paraspinal muscle	-0.57 (-0.67 to -0.45) *p* < 0.0001	-0.54 (-0.65 to -0.41) *p* < 0.0001
3T	pancreas:spleen	-0.40 (-0.58 to -0.19) *p* = 0.0003	-0.41 (-0.58 to -0.20) *p* = 0.0003
	pancreas:paraspinal muscle	-0.42 (-0.59 to -0.21) *p* = 0.0002	-0.43 (-0.60 to -0.22) *p* = 0.0001

CI = Confidence interval; ROI = Region of interest; SIR = Signal intensity ratio

### T1 SIR associations with demographic factors

**Fig. 4:**
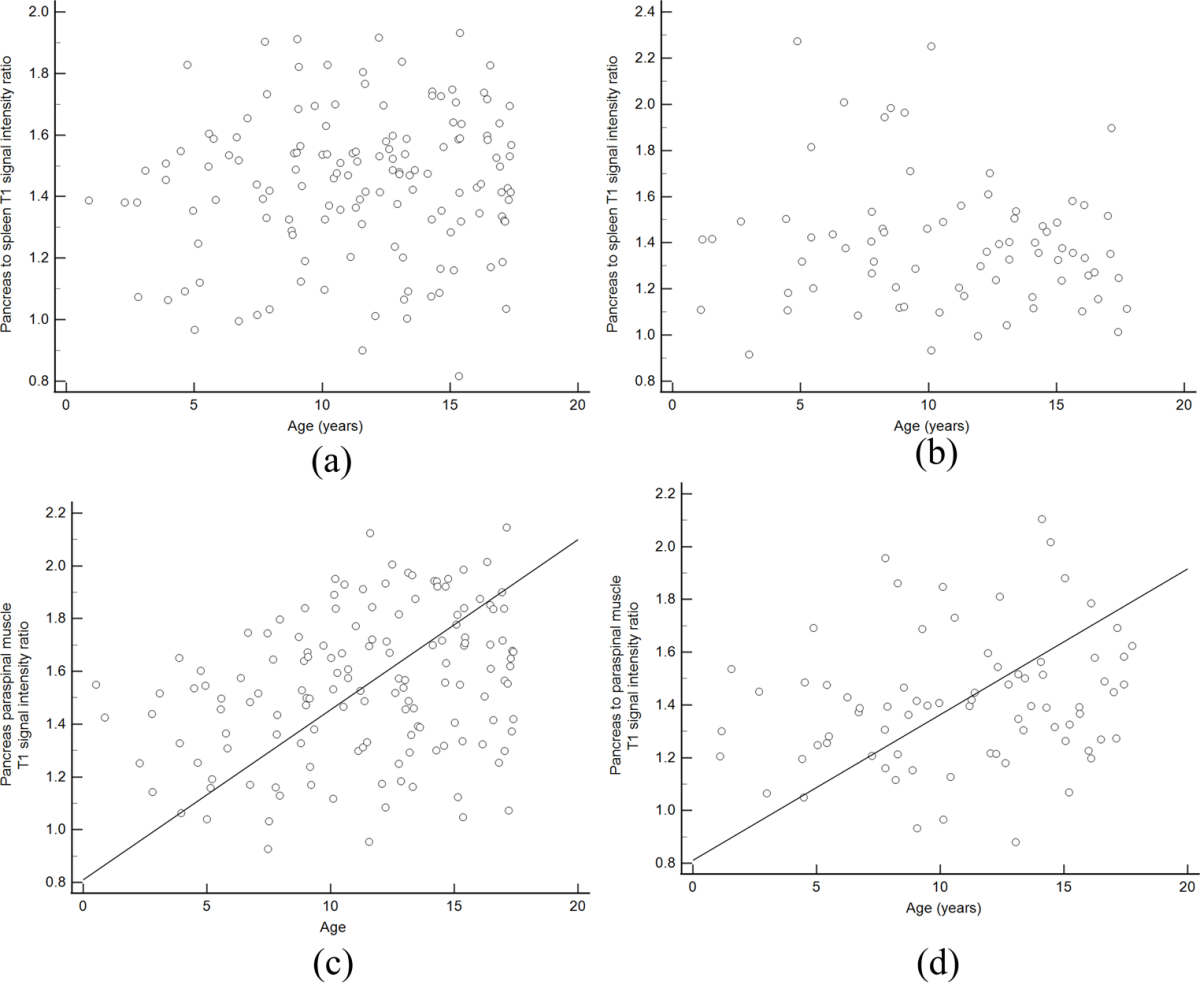
Scatter plots of T1 weighted signal intensity ratios (T1 SIR) versus age. For pancreas:spleen T1 SIR there was no significant correlation with age at **(A)** 1.5 T or **(B)** 3T (rho = 0.08 and rho=-0.09, respectively). For pancreas:muscle T1 SIR, there were weak but statistically significant correlations with age at both **(C)** 1.5T and **(D)** 3T (rho = 0.29 and rho = 0.24, respectively)

At 1.5 T there were no significant differences in T1 SIR-PS (*p* = 0.136) or T1 SIR-PM (*p* = 0.113) based on patient sex. There was no significant correlation between T1 SIR-PS and age (rho = 0.08; 95% CI: -0.09 to 0.24; *p* = 0.35) but there was a significant, weak strength correlation between T1 SIR-PM and age (rho = 0.29; 95% CI: 0.13 to 0.43; *p* = 0.0005) (Fig. 4). At 3T, T1 SIR-PS (*p* = 0.816) and T1 SIR-PM (*p* = 0.771) were not significantly different based on patient sex and there was no significant correlation between T1 SIR-PS and patient age (rho=-0.09; 95% CI: -0.31 to 0.14; *p* = 0.44). However, there was a statistically significant, weak strength positive correlation between T1 SIR-PM and patient age (rho = 0.24; 95% CI: 0.01 to 0.44; *p* = 0.04) (Fig. 4).

### T1 SIR differences based on magnetic field strength

T1 SIR-PS was not significantly different at 1.5T vs. 3T within patient groups. Similarly, SIR-PM showed no significant differences at 1.5T vs. 3T except in the group with no pancreatic disease where median T1-SIR was higher at 1.5T (1.65 vs. 1.46, *p* = 0.0016) (Table 3).

**Table 3:** Median T1 signal intensity ratios (SIR) for our study sample based on magnetic field strength of the scanner used. P-values reflect comparison of T1 SIR at 1.5T vs. 3T within diagnosis groups using Mann Whitney U test. Significant p-values are in bold

Patient Group	SIR-PS		SIR-PM	
	*1.5T*	*3T*	*1.5T*	*3T*
Acute pancreatitis	1.39 (1.17 to 1.54)	1.32 (1.12 to 1.41)	1.40 (1.30 to 1.56)	1.33 (1.16 to 1.50)
	*p* = 0.328		*p* = 0.374	
Acute on chronic pancreatitis	1.13 (1.07 to 1.29)	1.20 (1.13 to 1.45)	1.18 (1.12 to 1.46)	1.31 (1.20 to 1.45)
	*p* = 0.178		*p* = 0.556	
Chronic pancreatitis	1.44 (1.25 to 1.56)	1.36 (1.31 to 1.67)	1.52 (1.31 to 1.67)	1.37 (1.31 to 1.56)
	*p* = 0.445		*p* = 0.430	
No pancreatic disease	1.52 (1.40 to 1.64)	1.46 (1.32 to 1.64)	1.65 (1.49 to 1.84)	1.46 (1.30 to 1.65)
	*p* = 0.272		*p* **=** **0.0016**	

SIR-PM = signal intensity ratio of pancreas:paraspinal muscle; SIR-PS = signal intensity ratio of pancreas:spleen

### T1 SIR differences between patient groups

**Fig. 5:**
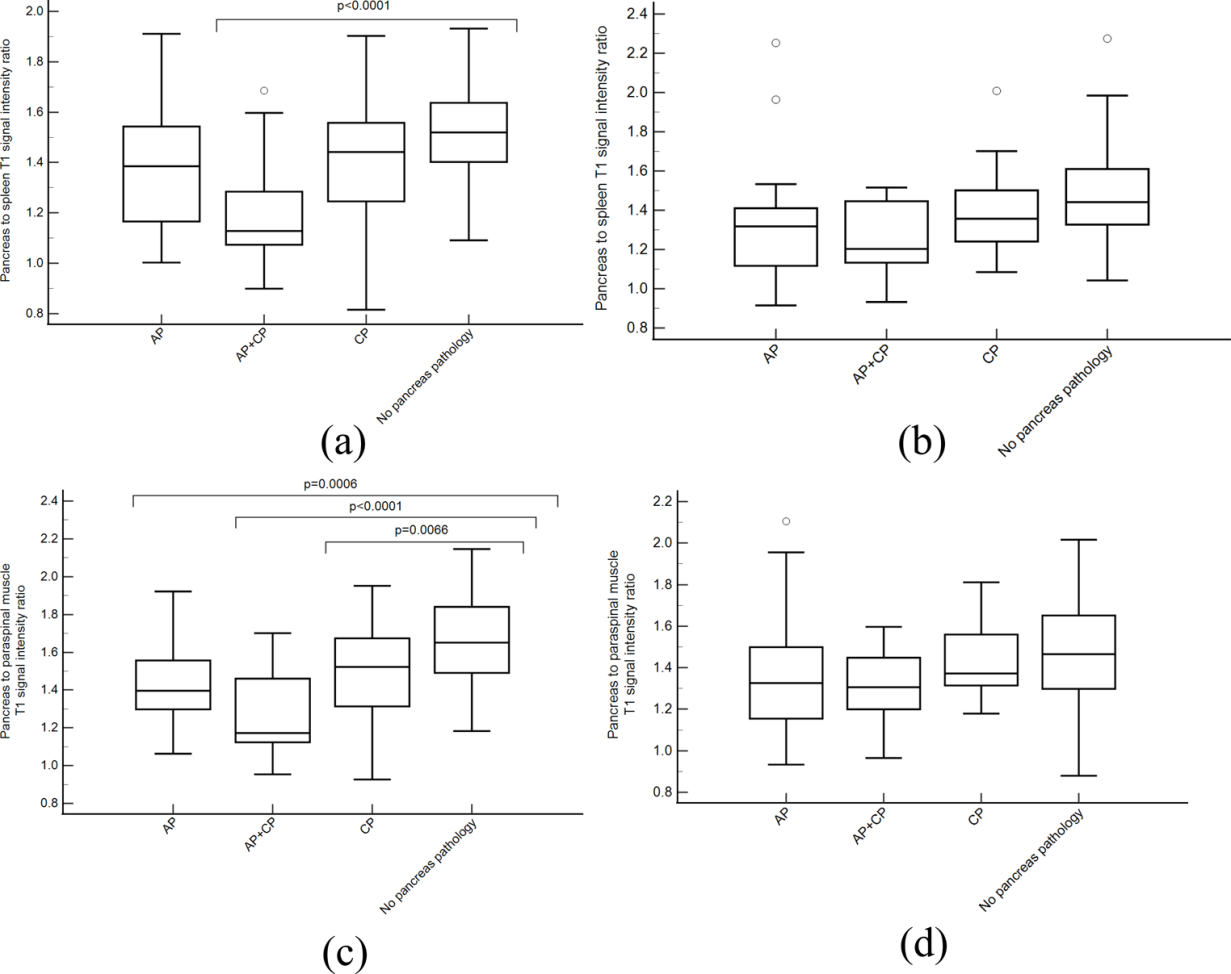
Boxplots of pancreas to spleen T1 weighted signal intensity ratio (T1 SIR-PS) at **(A)** 1.5T and **(B)** 3T and pancreas to paraspinal muscle T1 weighted signal intensity ratio (T1 SIR-PM) at **(C)** 1.5T and **(D)** 3T based on pancreatic diagnosis. Outliers are indicated by circles. Brackets indicate significant pairwise comparisons between no disease group and other groups (if present)

There were significant differences in T1 SIR-PS between patient groups at 1.5T (*p* < 0.0001) (Table 4; Fig. 5). Significant pairwise differences included: no pancreas pathology vs. acute on chronic pancreatitis (*p* < 0.0001) and chronic pancreatitis vs. acute on chronic pancreatitis (*p* = 0.0028). T1 SIR-PM was also significantly different between groups at 1.5T (*p* < 0.0001) with significant pairwise differences between: no pancreas pathology vs. acute pancreatitis (*p* = 0.0006), no pancreas pathology vs. acute on chronic pancreatitis (*p* < 0.0001), no pancreas pathology vs. chronic pancreatitis (*p* = 0.0066). Patients with acute or chronic pancreatitis had lower T1 SIRs than patients with no pancreas pathology but there were no significant differences in T1 SIR between the acute and chronic pancreatitis groups (*p* = 0.458 for T1 SIR-PS, *p* = 0.290 for T1 SIR-PM).

**Table 4:** Summary statistics for pancreas:spleen and pancreas:muscle T1 weighted signal intensity ratios (T1 SIR) based on diagnosis grouping at field strengths of 1.5T and 3T. Values are presented as medians and interquartile ranges (IQR). Pairwise comparisons were performed using Dunn’s test if Kruskal–Wallis test was statistically significant

Field strength	T1 SIR type [*n*]	Diagnosis Group	T1 SIR (IQR)	Significant (*p* < 0.05) pairwise difference	*P* value from Kruskal-Wallis
1.5T	Pancreas:spleen [140]	AP	1.39 (1.17 to 1.54)	None	< 0.0001
		AP + CP	1.13 (1.07 to 1.29)	CP (*p* = 0.0028) No pancreas pathology (*p* < 0.0001)	
		CP	1.44 (1.25 to 1.56)	AP + CP (*p* = 0.0028)	
		No pancreas pathology	1.52 (1.40 to 1.64)	AP + CP (*p* < 0.0001)	
	Pancreas:paraspinal muscle [143]	AP	1.40 (1.30 to 1.56)	No pancreas pathology (*p* = 0.0006)	< 0.0001
		AP + CP	1.18 (1.12 to 1.46)	No pancreas pathology (*p* < 0.0001)	
		CP	1.52 (1.31 to 1.67)	No pancreas pathology (*p* = 0.0066)	
		No pancreas pathology	1.65 (1.49 to 1.84)	AP (*p* = 0.0006) AP + CP (*p* < 0.0001) CP (*p* = 0.0066)	
3T	Pancreas:spleen [75]	AP	1.32 (1.12 to 1.41)	None	0.033
		AP + CP	1.20 (1.13 to 1.45)	None	
		CP	1.36 (1.31 to 1.67)	None	
		No pancreas pathology	1.46 (1.32 to 1.64)	None	
	Pancreas:paraspinal muscle [76]	AP	1.33 (1.16 to 1.50)	N/A	0.144
		AP + CP	1.31 (1.20 to 1.45)		
		CP	1.37 (1.31 to 1.56)		
		No pancreas pathology	1.46 (1.30 to 1.65)		

SIR = signal intensity ratio; AP = acute pancreatitis; AP + CP = acute on chronic pancreatitis; CP = chronic pancreatitis

At 3T, T1 SIR-PS was significantly different between patient groups (*p* = 0.033) but without significant pairwise differences. T1 SIR-PM was not significantly different between groups at 3T (*p* = 0.144).

### Receiver operating characteristic curve analysis

At 1.5T, the area under the receiver operating characteristic curve for distinction of patients with any pancreatitis vs. no pancreatic disease was 0.71 (95% CI: 0.63 to 0.79) for SIR-PS and 0.75 (95% CI: 0.67 to 0.82, *p* < 0.0001) for SIR-PM. Based on the Youden index, an SIR-PS cut-off of ≤ 1.31 had 44% sensitivity (95% CI: 31–57) and 95% specificity (95% CI: 87–99) and a SIR-PM cut-off of ≤ 1.53 had 69% sensitivity (95% CI: 56–80) and 70% specificity (95% CI: 58–80).

## Discussion

We have shown that in children, pancreas:spleen T1 SIR moderately to strongly correlates with estimated T1 relaxation time at 1.5T. At both 1.5T and 3T, pancreas:paraspinal muscle T1 SIR moderately correlates with estimated T1 relaxation time. These results suggest that this more available quantitative metric of T1 signal change could serve as a surrogate for more involved methods of estimating T1 relaxation time, providing a means to quantify T1 signal change for centers without access to T1 mapping techniques. The T1 SIR method also presents an opportunity to quantify pancreatitis-related T1 signal change using free-breathing (e.g. radial) imaging techniques. We have also shown that there are significant differences in T1 SIR based on the presence of pancreatic disease, especially between patients with acute or chronic pancreatitis and patients with no pancreas disease. Patients with pancreatitis generally have lower T1 SIR than patients without pancreatic disease and, based on ROC analysis, at 1.5T, thresholds of ≤ 1.31 for SIR-PS and ≤ 1.53 for SIR-PM have moderate performance for distinguishing patients with pancreatitis from patients without pancreatic disease.

Our results show less strong correlations between T1 SIR and estimated T1 relaxation time at 3T. Further, while median T1 SIR values were not significantly different between 1.5T and 3T, except for lower T1-SIR-PM values in patients without pancreatic disease at 3T, there were less pronounced differences in T1 SIR between patient groups in our sample at 3T. While these results are highly likely confounded by our relatively smaller sample size at 3T, there also appears to be some age dependence of pancreas:muscle T1 SIR that could influence the performance of that measure. In total, these findings suggest further study is necessary to understand T1 SIR more completely as a marker of pancreatitis in children at 3T.

T1 relaxation time has been shown to be prolonged in adults with chronic pancreatitis and is associated with the severity of acinar cell loss [18, 19]. Similarly, pancreatitis in children has been shown to be associated with longer T1 relaxation times compared to children without pancreatic disease [7]. These results suggest that estimated T1 relaxation time has a role as a non-invasive marker of pancreatic disease. However, estimating T1 relaxation time currently requires application of specific MR sequences (e.g., MOLLI) which require multiple breath holds, limiting applicability and availability in children. Further, T1 relaxation time is field strength dependent requiring different thresholds for normal vs. abnormal at 1.5T vs. 3T. Our results demonstrate that at 1.5T, the T1 SIR which can be obtained from more routinely performed T1-weighted imaging both correlates well with estimated T1 relaxation time and shows differences between patient groups, like differences previously demonstrated for T1 relaxation values.

Pancreas T1 SIR has been shown in other studies to stratify groups of adults with pancreatitis. Tirkes et al. reported a threshold value of 1.2 for T1 SIR-PS to have high sensitivity (77%) and specificity (88%) for exocrine dysfunction in adults with suspected chronic pancreatitis imaged at 1.5T [20]. Patients with a normal bicarbonate concentration in secreted pancreatic fluid had a mean T1 SIR-PS of 1.41 while patients with a low bicarbonate concentration in secreted pancreatic juice had a mean T1 SIR-PS of 1.03 [20]. In another multicenter study of adults, which used both 1.5T and 3T scanners, the T1 SIR-PS for the reference (no disease) group was 1.34 and the T1 SIR-PS for patients with different stages of pancreatitis ranged from 1.05 to 1.27 [9]. The same study also reported the SIR-PM for patients without disease to be 1.28 and for patients with different mechanistic stages of chronic pancreatitis to range between 0.95 and 1.17 [9]. These T1 SIR values are lower than those observed in both our pediatric patients with no pancreas pathology (median SIR-PS = 1.52; median SIR-PM = 1.65) and our pediatric patients with chronic pancreatitis (median SIR-PS = 1.44; median SIR-PM = 1.52) and threshold values for T1-SIR derived from our sample are higher (≤ 1.31 for SIR-PS and ≤ 1.53 for SIR-PM). Applying the SIR-PS threshold of 1.2 defined by Tirkes et al. to our sample would produce low sensitivity (36%) but high specificity (96%) [20]. The mean T1 SIR-PS previously reported for children without pancreatic disease imaged at 1.5T by McCleary et al. is also lower than seen in our sample at 1.32 (reader 1) and 1.33 (reader 2) [12]. These differences in SIR values may reflect differences in the specific T1W sequence used to make SIR measurements and suggest the need for comparative study of the impact of sequence parameters on pancreas T1 SIR.

Our study is limited by its retrospective design and the fact that we only analyzed images from a single MR manufacturer, obtained at a single institution. Because of this limitation, our results may not be immediately generalizable to other scanner manufacturers without validation. Further, the relatively lower sample size at 3T likely impacts our results and precluded ROC analysis at 3T. Additionally, ROIs were drawn by only one observer but with confirmation of positioning by a pediatric radiologist. This precludes quantification of interobserver variation in measured T1 SIR. While assessment of interobserver variation in T1 SIR measurement was not the focus of this study, any such variation might impact the strength of correlation with estimated T1 relaxation time and might impact the degree of difference between patient groups. Finally, while we observed significant differences in T1 SIR between patient groups, because ratios are being used, these differences are relatively small.

While our results suggest potential for T1 SIR as a quantitative measure of pancreatitis-related T1 signal change, future studies based on data from multiple MRI scanners (manufacturer and field strength) with multiple observers making measurements will be useful to validate and build on our findings. Additionally, studies exploring the role of ROI standardization (size, shape, position) will be important to optimize implementation of T1 SIR.

## Conclusion

Pancreas to spleen and pancreas to muscle T1 SIR are strongly and moderately correlated with T1 relaxation time estimates and are significantly lower in children with pancreatitis (acute and/or chronic) at 1.5T. At 3T, the strength of correlation is moderate and significant differences between patient groups were less apparent. Our results suggest that the easily obtainable T1 SIR can be used to identify pancreatitis-related changes in T1 signal at 1.5T but that more study is needed regarding its use at 3T. We encourage routine reporting of the T1 SIR for MR imaging performed for pediatric pancreatitis.

## Data Availability

The data that support the findings of this study are available from the corresponding author upon reasonable request.
